# Complete Genome Sequence of *Gluconacetobacter hansenii* Strain NQ5 (ATCC 53582), an Efficient Producer of Bacterial Cellulose

**DOI:** 10.1128/genomeA.00785-16

**Published:** 2016-08-11

**Authors:** Sarah Pfeffer, Kalpa Mehta, R. Malcolm Brown

**Affiliations:** University of Texas at Austin, Molecular Biosciences, College of Natural Sciences, Austin, Texas, USA

## Abstract

This study reports the release of the complete nucleotide sequence of *Gluconacetobacter hansenii* strain NQ5 (ATCC 53582). This strain was isolated by R. Malcolm Brown, Jr. in a sugar mill in North Queensland, Australia, and is an efficient producer of bacterial cellulose. The elucidation of the genome will contribute to the study of the molecular mechanisms necessary for cellulose biosynthesis.

## GENOME ANNOUNCEMENT

The Gram-negative bacterium *Gluconacetobacter hansenii* (formerly *Acetobacter xylinum*) is a particularly efficient producer of pure, highly crystalline cellulose known as bacterial cellulose (BC) (1–3). Because of its ultrafine reticulated structure, high crystallinity, great mechanical strength, high water-holding capacity, moldability during formation, and biocompatibility, BC is well suited for medical, industrial, and commercial applications (4–7). These qualities make BC a potentially useful biomaterial. The results presented in this report will provide important insight into the molecular mechanisms of bacterial cellulose biosynthesis and will add to the study of the *Gluconacetobacter* genus.

We have sequenced the genome of *G. hansenii* strain NQ5 (ATCC 53582). This strain is a highly efficient producer of BC and has been used for a range of studies on the effects of growth conditions on cellulose biosynthesis (7–10). However, until now, the genome of this organism had not been available for study. *G. hansenii* strain NQ5 was isolated by R. Malcolm Brown, Jr. from a sugar mill in North Queensland, Australia, in 1981. Its DNA was extracted and subjected to sequencing using the Illumina HiSeq 2500 sequencer (University of Texas at Austin, ICMB Core Facility). The reads were assembled into contigs using Velvet version 1/2/02 (11) and downloaded into Geneious version 8.1.2 (12), and open reading frames (ORFs) were predicted using Glimmer (13). Preliminary annotation data on contigs containing cellulose synthase genes were determined.

The DNA sequence of *G. hansenii* strain NQ5 was resolved, and bioinformatics analysis revealed that it is approximately 3.38 Mbp in size with a GC content of 55.6%. A total of 6,839 ORFs were identified in the genome (13). The complete annotation of the full genome is in progress.

Preliminary annotation indicated that *G. hansenii* NQ5 contained three cellulose synthase-coding regions identified as *acsABCD*, *acsAII*, and *acsABC*. A homology comparison for cellulose synthase was performed, and it was determined that *G. hansenii* ATCC 23769 (GenBank accession no. AB091060) has a 99% sequence similarity to all of the regions coding for cellulose biosynthesis (Geneious, MUSCLE, ClustalW, and MAFFT). No other strain of *Gluconacetobacter* sp. shared this sequence similarity or contained three cellulose synthase-coding regions. It is important to note that the *acsABCD* operon is flanked by genes coding for proteins that have been determined to be essential for proper cellulose biosynthesis to occur: *cmcAx*, *ccpAx*, and *bglAx* (14–17). Additionally, these flanking genes also shared 100% homology.

Further studies into third-region coding for cellulose biosynthesis and how it may or may not impact cellulose production could provide valuable insight into the important biological systems needed for cellulose biosynthesis.

### Accession number(s).

This whole-genome shotgun project has been deposited in DDBJ/ENA/GenBank under the accession number LUCJ00000000. The version described in this paper is the first version, LUCJ01000000.
